# An Account of the Medicinal Properties, &c. of the Kava Shrub (Piper Methisticum), Gambir (Nauclea Gambir), and the Ignatia Amara, or St. Ignatius' Bean

**Published:** 1832-02

**Authors:** George Bennett

**Affiliations:** Corresponding Member of the Medico-Botanical Society of London.


### MEDICINAL PROPERTIES OF THE KAVA SHRUB, &c.

*An Account of the Medicinal Properties, &c. of the Kava Shrub (Piper Methisticum), Gambir (Nauclea Gambir), and the Ignatia Amara, or St. Ignatius' Bean.* By GEORGE BENNETT, F.L.S., M.R.C.S., Corresponding Member of the Medico-Botanical Society of London.

(An Abstract of a Paper read before the Medico-Botanical Society of London, January 10th, 1832.)

### *The Kava, or Ava Shrub, of Polynesia.*

THE Kava, or Ava, (Piper Methisticum,) is one of the Polynesian shrubs, to which medicinal properties have been attributed, and in which some confidence has been placed by European and American practitioners. The root, prepared by mastication, and placed in a bowl, to which water is added, forms an intoxicating beverage, much in use among the Polynesian islands. At the island of Tongatabu (one of the Friendly group,) I observed two kinds: one termed the true Kava of the natives, and seen only in a cultivated state; the other was abundant, wild. The true Kava has a crooked, jointed stalk, rising to the height of eight or nine feet; the leaves are heart-shaped, pointed, many-nerved, large, and of a dark-green colour; the spikes are axillary and solitary; and the fruit, when mature, of a bright red colour. The other species is called Kava, kava ului by the natives, and is seen growing abundantly in a wild state: it does not attain the height, nor has it the thickness of stalk, of the true Kava; the leaves are orbicular, heart-shaped, and of a bright shining green colour; it resembles the Piper latifolium of Forster, excepting that my specimens have solitary spikes, whilst those of the specimens in Sir Joseph Banks' Herbarium (deposited in the British Museum,) are aggregate. This species is not used by the natives for any purpose. Among the Fidgis, Friendly, and Navigator's group of islands, &c. a root of the Kava plant is presented as an emblem of peace.

The root of this shrub is considered as an efficacious remedy in cutaneous disorders, or affections of the mucous membranes: it has been frequently used, I was informed, in the United States; and an American gentleman, whom I met at the Sandwich islands, informed me that he had been long suffering from erysipelas, which was cured by an infusion of this root twice daily, when the disease would yield to no other remedy. An infusion of this root has also been used, and found beneficial in gonorrhœa.

During my visit to the Sandwich islands, in November 1829, Governor Boki informed me that the Kava root varied very much in its quality, which rendered some much better calculated for medicinal purposes than others: he presented me with specimens of the best quality, and it still retains its peculiar smell and flavour. It is said that too great a degree of heat will destroy the virtues of this root; it should consequently be prepared thus: After the root has been well bruised, it should be infused for about twenty-four hours in tepid water; half a pint of the infusion to be given every night and morning.* Mr. Collie, surgeon of the Blossom, observes, that "a course of it is most beneficial in renovating constitutions which have been worn out by hard living, long residence in warm climates, (without, however, affections of the liver,) and by protracted chronic diseases; more especially if the disorder be such as by the humoral pathologists would be attributed to an attenuated or acrid state of the blood."

*The Nauclea Gambir, producing the Gambir, Catechu, or Terra Japonica of commerce.*

The Nauclea Gambir is placed by Jussieu under the natural order Rubiaceæ: it is a shrub attaining the height of six to eight feet, branchy; the leaves are ovate, pointed, smooth, waving, distinctly veined transversely underneath, of a dark green colour, and, when chewed, they have a bitter astringent taste, leaving, however, afterwards a sweetish taste in the mouth, not unlike liquorice; the flowers are aggregate, globular, composed of numerous florets, crowded on a globular, naked receptacle; tubes of the corolla of a pinkish colour; the upper part of the corolla fine, cleft, and of a greenish yellow colour; the stamina are five in number, and short; the pistil is longer than the corolla; the flowers are destitute of fragrance; the capsules (as correctly stated by Mr. Hunter,) "are stalked, oblong, incrusted and crowned with the calyx; tapering to a point below; two celled, two valved, the valves adhering at the apex, splitting at the sides; seeds very numerous, oblong, very small, compressed, furnished at both ends with a membranous pappus."

From observations made at Singapore, I am induced to consider the tree as diœcious, from observing numerous trees, among which some were in full flower, of which the corolla falls off, leaving the calyx, which withers without any appearance of the ovarium becoming perfect; others were covered with immature and mature capsules; but the fertile appearance of the stigma in the specimens I collected would

* Half an ounce of the root is a dose, macerated in a proportionate quantity of water.

cause me, in some degree, to doubt the fact of its being diœcious: I, however, mention the circumstance for future investigation. The shrubs also I observed at Singapore were not climbing.

This shrub yields the Gambir, Terra Japonica, or Catechu* of commerce, and is an extract prepared from the leaves; a catechu is also prepared in India from a species of acacia (A. catechu,) which is found growing plentifully in Hindoostan, on the mountains of Kanhana; and there are also two kinds said to be produced from the nut of the Areka palm, named in India Cattacamboo and Cashcutti, and both are used by the Indian practitioners.†

Its medicinal properties are astringent, and it is considered useful in diarrhœa and dysentery, in gleet, catarrhal affections, &c. Alkaline salts destroy its astringent powers, and metallic salts and solution of isinglass are incompatibles. The dose is usually from twelve grains to one drachm.

The Gambir shrub is propagated either by seeds or cuttings, but the latter are preferred. It was formerly cultivated to some extent at Singapore, (where I had an opportunity of observing it in November 1830,) but the cultivation of the shrub and preparation of the extract is now neglected; the reason assigned for which was, that the Gambir can be imported cheaper from the islands in the vicinity, more especially at the Dutch settlement at Rhio: a smaller quantity, however, is grown by some of the Chinese settlers for their own immediate consumption, but not so extensively as to form an article of commerce.

The extract is used extensively by the natives of India, Eastern Archipelago, Cochin China, and Cambodia, as a masticatory, wrapped up with the betel. There are different qualities of extract: the first and best is white, brittle, and has an earthy appearance when rubbed between the fingers, (which earthy appearance gave it the name of Terra Japonica, being supposed at first also to come from Japan,) and is formed into very small round cakes. This is the dearest kind, and most refined, but it is not unfrequently adulterated with sago: this kind is brought in the greatest quantity from the island of Sumatra.

The second quality is of a brownish yellow colour, is formed into oblong cakes, and, when broken, has a light brown, earthy appearance; it is also made into a solid cube form: it is sold in the bazaars in small packets, each containing five or six.

* *Kate* signifies a tree, and *Chu* juice, in the Oriental language.

† Thomson's Dispensatory, p. 129.

The third quality contains more impurities than the preceding, is formed in small circular cakes, and is sold in packages of five or six in the bazaars.*

The method employed in preparing the extract is thus correctly related by FINLAYSON: "The leaves are collected three or four times a year; they are thrown into a large cauldron, the bottom of which is formed of iron, the upper part of bark, and boiled for five or six hours, until a strong decoction is obtained; the leaves are then withdrawn, and allowed to strain over the vessel, which is kept boiling for as many hours more, until the decoction is inspissated; it is then allowed to cool, when the catechu subsides. The water is drawn off; a soft soapy substance remains, which is cut into large masses; these are further divided by a knife into small cubes, about an inch square, or into still smaller pieces, which are laid in frames to dry. This catechu has more of a granular, uniform appearance than that of Bengal: it is, perhaps, also less pure."†

A Gambir manufactory is usually observed near a pepper plantation, as the pepper vine does not thrive in the soil of Singapore unless well manured: the refuse of the leaves, &c. used in the manufacture of the extract is found excellent for the purpose of manuring the vines.

The younger leaves of the shrub are said to produce the whitest and best Gambir: the older, a brown and inferior sort. There are other species of Nauclea indigenous to Singapore, but they do not produce any extract.

*The Ignatia Amara, or St. Ignatius' Bean: its Medicinal Uses among the Natives of the Philippine Islands, &c.*

The tree said to produce the St. Ignatius' bean‡ is placed in the natural order Luridæ of Linnæus, Apocineæ of Jussieu, class Pentandria, order Monogynia. As a medicinal remedy it is in great repute in Western India, and it is commonly known throughout India by the name of Papeeta. It was regarded as a valuable remedy in spasmodic cholera, but repeated trials made in India did not justify its being classed as a specific in that disease.

The tree is indigenous to the Philippine islands, Cochin China, &c.; at the former group of islands, it is more usually found growing in the islands of Samar, Leyte, Zebu, Panay, &c., and is brought from thence to Manilla, where

* Specimens of the different kinds were laid before the Society.

† Finlayson's Mission to Siam and Hué, p. 56, 7.

‡ This appellation is apt to mislead: it has not any affinity to the leguminous tribes.

the fruit is sold in the bazaars, and is much used by the natives, both as an external and internal remedy.

The tree is not well known to botanists: it has been described by LOUREIRO under the name of Ignatia Philippinica, and who describes it "as a large shrub with a trunk like a tree, and many very long, climbing, unarmed branches; leaves large; the tube of the corolla reclining; the fruit a large roundish berry, attenuated at the neck, juiceless; the rind very smooth, woody, whitish, like the bottle gourd when ripe. Seeds three-cornered, ovate, brownish ash colour, smooth, of a horny substance, and very bitter taste." The younger LINNÆUS describes it as a "very branching tree; the branches long, round, very smooth, climbing; leaves opposite, petioled, a span long, quite entire, veined, very smooth, flat; flowers in small panicles, peduncles bearing from three to five flowers; pedicles short, round, rigid; flowers very long, nodding, white, having the smell of jasmine; fruit ovate, with a very smooth, dry rind, narrowed at the neck, the size of a Bronchretian pear."

These are the only accounts, I believe, we have of the tree, on making repeated inquiries during my visit to Manilla. I could not see it, as it was not found growing in any of the districts about Manilla, but only on the before-mentioned islands forming the Philippine group: the natives described it as attaining a great height. The fruit can be purchased in any quantity in the bazaars at Cavité or Manilla, at a very cheap rate.* Taking a description from those specimens, the fruit is large, round, externally of a black colour and somewhat wrinkled appearance; on breaking the shell, it is found lined internally with a dense, tough substance; sometimes the seeds were found sacculated around the internal part of the shell, and at others clustered together; in one instance I found them imbedded in a glutinous, blackish pulp; the number of seeds in each capsule varies from one to twelve; they are tetragonal in shape, and covered by a light brown epidermis, which easily rubs off, and are then of a blackish colour, internally whitish, and of a horny consistence.

The medicinal properties ascribed to the seeds are tonic, diaphoretic, anthelmintic; it is also stated to be used in colic, intermittent fevers, &c. At Manilla, it is called Cabalonga by the natives, and it is used by them in spasmodic pains of the bowels and other visceral complaints, and is

* Four of the pods for a real was the rate at which I purchased them, and I, no doubt, paid a high price as a foreigner.

also considered as an excellent tonic: it is used both as an internal remedy, and applied, mixed with oil, externally. It is prepared in the following manner for external application: The nut is rubbed down with vinegar, until a kind of paste is formed; it is then spread as a plaster, and, if the pain is in the head, a plaster is placed on the temporal region, and it is said soon to alleviate the most violent pain; it is also mixed with oil, and rubbed on the affected part. As an internal remedy, it is given rubbed down with vinegar, or in the form of powder, in doses of half a grain to ten grains. The Spaniards and some of the natives, when using it as an internal remedy, prepare it in this manner: they take one of the nuts, and place it, for about the space of two minutes, in hot tea or coffee, when the nut is taken out, and the infusion drank. Wine is also placed in the calabash, or external shell of the capsules, (which has, like the seeds, a very bitter taste,) from which the seeds have been removed, and, being permitted to remain for eight or nine minutes, the wine, which has then acquired a bitter taste, is drank, and regarded as an excellent tonic. The native Indians also use it as an external application in bruises, pains of the head, rheumatism, &c. The fruit which is found to contain only one seed is considered the most valuable and the strongest, and requires a less dose, when administered, than of those seeds of which several were contained in one capsule: the seeds are said to be in great request as a medicine in Spain, and also in South America.

When an overdose was taken, violent pains in the head, vertigo, and tetanic symptoms are produced. I could not ascertain that any antidote was used among the natives: lemonade has been stated as one.

I tried experiments of the poisonous effects of the seeds in two instances; one on a dog about ten months old, and with the following results.

At fifty-five minutes past ten A.M., I gave half a drachm of the seed, cut into small pieces, and enveloped in a piece of meat, to a dog: it was swallowed without any of the pieces being lost.

It was lively and playful, but at times would appear restless and uneasy, as if some internal annoyance was occasioned by the poison. At twenty minutes past eleven, he suddenly fell with violent convulsion of the limbs, which were extended with great rigidity, and they afterwards remained in that rigid position: this was followed by excessive panting, and trembling of the muscles; the saliva became viscid, and the tongue, as well as the saliva with which it was covered, had a dark appearance. The poison acted on the nervous system; the dog exhibited no indication of pain; no yell escaped him; the eyes assumed a dull appearance; the mouth had a movement as if the animal had been snapping at flies; and there was a constant spasmodic twitching of the muscles of the face.

At twenty-five minutes past eleven, the body became less convulsed; the urine was passed involuntarily, but not the fæces. On passing the hand before the eyes of the animal, the eyelids were moved, indicating that consciousness had not yet departed; the orbicularis oculi muscles had a convulsive quivering; the snapping and panting still continued.

At thirty-five minutes past eleven, a general convulsive action of the muscles came on, and terminated the existence of the animal.

An hour after the death of the animal I inspected the body. On opening the thorax, the viscera had no particular appearance. On inspecting the stomach, it was found partly filled with rice, among which the small pieces of the seeds of the Ignatia amara were intermingled: on emptying the stomach of its contents, the inner coat was found of a pale pinkish hue; the liver had a healthy appearance, but, if any portion of that organ was cut, a quantity of blood in a fluid state immediately flowed; and it may be an interesting question, why we find the blood destitute of fibrin in those cases in which death proceeds from causes acting immediately on the nervous system, as where a person is struck dead by lightning, &c.* The remainder of the abdominal and pelvic viscera had a healthy appearance; the whole of the muscles of the animal had a bloodless, blanched appearance; the vena cava and aorta were also filled with blood in a fluid state. On inspecting the brain, no particular appearance was observed, excepting that the vessels appeared totally destitute of blood.

The second experiment was on a dog about a year and a half old.

At thirty-five minutes past four P.M., half a drachm of the seed, cut into small pieces, was given to the animal.

At five minutes past five, he suddenly fell with violent spasms of the limbs, which soon became stretched out and rigid, with spasms of the lower jaw. I was desirous of trying vinegar as an antidote; so, as soon as the spasm of the muscles of the lower jaw relaxed, I poured down some

* This deficiency of fibrin in the blood is also found in dropsical persons after death.

quantity:* the animal at this time appeared nearly dead. About two minutes after the vinegar had been administered, he was so much recovered as to stand up: it was, however, but of short duration, for, at ten minutes past five, he reeled and fell with similar spasms as before. Another dose of vinegar was given, with the same good effects: the animal recovered, and stood up; the eyes, however, preserved throughout a dull, glairy appearance; the dog appeared uneasy, as might have been expected, and trembled exceedingly, but no expression of pain escaped him; he seemed bewildered.

At fourteen minutes past five, he fell, with similar effects as before; the spasms were not, however, so severe: the same remedy was given, with the same reviving effects. The fits were accompanied at first by violent spasmodic action of the muscles of the lower extremities, which afterwards became extended and rigid, whilst other parts of the body were convulsed. The muscles of the jaw had invariably a strong spasmodic action at the commencement of the fit, but soon became relaxed. The panting and snapping were not so marked in this as in the preceding experiment.

At eighteen minutes past five, he again fell; the urine was passed involuntarily, but not the fæces, and he appeared nearly dead: vinegar was again administered, but he lay for some time without hardly an indication of existence. I thought life was extinct. At twenty-five minutes past five, the respiration, before scarcely to be perceived, became laborious, as if the circulation of the blood had again taken place by a renewal of the heart's action; the eyes became animated, and the dog regained his sensibility: the laborious state of respiration only continued for a short period.

At thirty-five minutes past five, he had not risen from his extended posture on the ground, but no fit had again yet taken place. On causing him to be raised from the ground, the limbs displayed a contracted, rigid appearance, but they could be readily placed by the hand in a relaxed position. He was soon afterwards able to sit up by his own exertions: he was then so far recovered as to run the length of the cord with which he was tied, but the limbs, when he moved, appeared stiffened and paralytic. The animal, a short time previous to his being brought to me, had received an injury of the left hind leg, which had occasioned lameness: when the spasmodic action of the muscles came on during the

* About a minute had elapsed from the time of the poison taking effect to the administration of the vinegar as an antidote.

paroxysms, the injured limb was drawn upwards and backwards, whilst the other limbs were drawn stiffened downwards and outwards.

The animal continued apparently well, but in a miserable debilitated state, until fifty minutes past five, when he moved about, and fell down suddenly with the same tetanic symptoms as before; the eyes had again their dull, glairy appearance; the jaws were kept firmly closed by the violence of the spasms, which severe spasms were extended over the whole of the body, and in the space of a minute he was dead. The quantity of vinegar taken was about half a pint.

The poisonous effects of these seeds are speedy and violent. Magendie considers that its active principle, the strychnine, strongly excites the spinal marrow, without affecting, except indirectly, the functions of the brain. After the dog has taken the poison, his playful manner still continues, but mixed at the same time with a restlessness, which increases as the poison begins to act; he then feels inclined to lie down, then rises again, until he suddenly falls with tetanic symptoms, and during the action of the poison the animal appears bewildered.

The active principles of the Ignatia amara are strychnine and brucine, of which the first is the most powerful, and is found purer in the ignatia than in the nux vomica, but purest in the Upas tieuté of Java.

According to the analysis of MM. Dumas and Pelletier, the mean of strychnine in a hundred parts is

| | |
|---|---|
| Carbon . . . . . | 78.22 |
| Azote . . . . . . . | 8.92 |
| Hydrogen . . . . . | 6.54 |
| Oxygen . . . . . | 6.38 |
| | 100.06 |

It has been stated "as unfortunate that the bean of St. Ignatius is so rare an article of commerce, as the strychnine contained in it is nearly free from brucine, and would readily be obtained from it in a state of purity." At the Philippine islands it can be procured in any quantity, at a very cheap rate: it forms a commercial article from Manilla (the capital of the group,) to Spain and South America.

In this country (in the form of strychnine) it has now become a valuable remedy in paralysis unattended by organic disease of the brain, more especially in those cases occasioned by lead.

*London; January 20th, 1832.*

